# Annual Meeting of the International Society of Cancer Metabolism (ISCaM): Metabolic Adaptations and Targets in Cancer

**DOI:** 10.3389/fonc.2019.01332

**Published:** 2019-11-28

**Authors:** Sofia Avnet, Nicola Baldini, Lucie Brisson, Stine Falsig Pedersen, Paolo E. Porporato, Pierre Sonveaux, Gyorgy Szabadkai, Silvia Pastorekova

**Affiliations:** ^1^Orthopaedic Pathophysiology and Regenerative Medicine Unit, IRCCS Istituto Ortopedico Rizzoli, Bologna, Italy; ^2^Department of Biomedical and Neuromotor Sciences, University of Bologna, Bologna, Italy; ^3^INSERM UMR1069, Nutrition, Croissance et Cancer, University of Tours, Tours, France; ^4^Department of Biology, Faculty of Science, University of Copenhagen, Copenhagen, Denmark; ^5^Department of Molecular Biotechnology and Health Science, Molecular Biotechnology Center, University of Torino, Turin, Italy; ^6^Pole of Pharmacology and Therapeutics, Institut de Recherche Expérimentale et Clinique, Université Catholique de Louvain, Brussels, Belgium; ^7^Department of Biomedical Sciences, University of Padua, Padua, Italy; ^8^Department of Cell and Developmental Biology, Consortium for Mitochondrial Research, University College London, London, United Kingdom; ^9^The Francis Crick Institute, London, United Kingdom; ^10^Biomedical Research Center, Slovak Academy of Sciences, Bratislava, Slovakia

**Keywords:** cancer metabolism, proton dynamics, tumor microenvironment, cancer imaging, cancer therapy

## Abstract

The metabolism of cancer cells differs from that of their normal counterparts in a spectrum of attributes, including imbalances in diverse metabolic arms and pathways, metabolic plasticity and extent of adaptive responses, levels, and activities of metabolic enzymes and their upstream regulators and abnormal fluxes of metabolic intermediates and products. These attributes endow cancer cells with the ability to survive stressors of the tumor microenvironment and enable them to landscape and exploit the host terrain, thereby facilitating cancer progression and therapy resistance. Understanding the molecular and physiological principles of cancer metabolism is one of the key prerequisites for the development of better anticancer treatments. Therefore, various aspects of cancer metabolism were addressed at the 5th annual meeting of the International Society of Cancer Metabolism (ISCaM) in Bratislava, Slovakia, on October 17–20, 2018. The meeting presentations and discussions were traditionally focused on mechanistic, translational, and clinical characteristics of metabolism and pH control in cancer, at the level of molecular pathways, cells, tissues, and organisms. In order to reflect major healthcare challenges of the current era, ISCaM has extended its scope to metabolic disorders contributing to cancer, as well as to opportunities for their prevention, intervention, and therapeutic targeting.

## Introduction

Research on cancer metabolism attracts an increasing interest, not only because of efforts to better understand principles of cancer progression but equally because of the need to bring new ideas and strategies for more effective anticancer therapies. Due to the enormous metabolic plasticity of cancer cells responding and adapting to signals from intracellular and extracellular milieu, this research field is very complex and rapidly evolving. To address challenges of this dynamic research area, the International Society of Cancer Metabolism (ISCaM) was founded in 2014 as a logical expansion of the International Society for Proton Dynamics in Cancer (ISPDC) that operated from 2009 to 2014. ISCaM's scope is to foster communication and collaboration between scientists engaged in research of metabolism, acidity, proton dynamics, and microenvironment in cancer (1–4). On October 17–20th 2018, ISCaM hold its 5th annual meeting in Bratislava, Slovakia. With a focus on “Metabolic Adaptations and Targets in Cancer,” this symposium brought together fundamental researchers and clinicians, scientists of international reputation and young investigators and ISCaM members and the international scientific community to exchange the most recent knowledge related to cancer metabolism. The meeting consisted of two keynote lectures and seven sessions with oral presentations by both senior speakers and junior researchers. The talks were accompanied by informative poster sessions. As an expression of high mutual trust among ISCaM members and other participants, many presentations included entirely new and unpublished data that underlined the meeting quality and the ISCaM value.

During the opening lecture, Adrian L. Harris (University of Oxford, UK) reported on new targetable aspects of cancer metabolism. He focused on mechanisms of cellular adaptation to glutamine deficiency, which is one of the key amino acids in cancer metabolism, and described a new pathway of metabolic reprogramming and regulating amino acid uptake in conditions of hypoxia-associated low glutamine.

## Metabolic Disorders and Cancer

Modern society generates a stressful and obesogenic environment promoting the development of metabolic disorders that contribute to many chronic diseases, including cancer. In the initial talk of this session, Stephan Herzig (Helmholtz Center Munich, Germany) explained the role of lipid metabolism in metabolic dysfunction-related cancer. He emphasized that obesity-associated complications do not only increase tumor risk but also promote metastatic tumor recurrence, thereby imposing a major threat on affected patients. In turn, end-stage tumor diseases have a significant impact on systemic metabolism, in particular causing body wasting, i.e., cachexia. He discussed the latest findings on key events and alterations of lipid metabolism relevant for both obesity-triggered metastasis formation and cancer cachexia, including their therapeutic aspects. Jozef Ukropec (Biomedical Research Center SAS Bratislava, Slovakia) then briefly reviewed data on the metabolic and functional flexibility of the adipose tissue, which largely determines the metabolic health of humans living in the obesogenic environment. Next, he described the skeletal muscle as an endocrine organ providing a spectrum of bioactive substances to orchestrate the whole-body adaptive response to exercise in recently finalized intervention studies. He also presented the design of a currently ongoing study aimed at investigating benefits of regular exercise in testicular germ cell cancer survivors. Elisabeth Wyart (University of Torino, Italy) focused on cachexia, which is a multi-organ wasting syndrome characterized by irreversible skeletal muscle atrophy that dramatically increases both morbidity and mortality in various diseases. Despite its high prevalence in cancer patients, the mechanism of cancer-induced cachexia remains unclear. So far, unpublished findings obtained by investigating cachectic mice bearing CT26-colon cancer suggest striking alterations in certain regulators of metabolism. Sofia Avnet (Rizzoli Orthopedic Institute, Italy) provided a thorough overview of the complex relationships between systemic/local acidosis and insulin resistance/signaling. Insulin influences acidosis by promoting glycolysis and, in turn, acidosis lowers insulin binding to its receptor, inhibits glycolysis, inhibits the recycling of glucose transporters and reduces insulin secretion by pancreatic β-cells via transforming growth factor β. Insulin also affects ion/proton transporters and pH sensors, and resulting acidosis may enhance insulin resistance through the NF-κB-driven inflammatory pathway. Eduardo Maldonado (Medical University of South Carolina, USA) presented the current view on the modulation of mitochondrial metabolism by statins in cancer cells, and suggested that it is not mediated by changes in cholesterol content and does not affect glycolysis. Overall, insights on the relationships between metabolic disorders and cancer provided in this session support the importance of continuing investigations on this topic, which is highly relevant for improving the lifestyle and healthcare of human populations.

## Genetic, Epigenetic, and Transcriptional Elements of Cancer Metabolism

In this session, the first presentation by Carlos Sebastian (Candiolo Cancer Institute, Italy) focused on the epigenetic control of tumor transformation in the intestinal tract. In APC^min^ mice, a model prone to intestinal and mammary cancer due to a non-sense mutation in an allele of the adenomatous polyposis coli (APC) gene, loss of NAD^+^-dependent histone deacetylase Sirtuin 6 (SIRT6) resulted in histone hyperacetylation, increased the abundance of intestinal stem cells (ISCs) and enhanced the tumor-initiating potential. Due to high pyruvate dehydrogenase kinase (PDK) activity, ISCs were highly glycolytic and had low ROS production, supporting stemness. After transformation, they were proposed to be at the origin of a Warburg-phenotype lineage of cancer cells in established intestinal tumors. Relationships between cancer cell metabolism and epigenetics were further highlighted by Thomas Grunt (Medical University of Vienna, Austria), who proposed systems biology as the best approach to decipher and target these networks. For example, he showed that fatty acid synthase (FASN) not only controls lipogenesis, but also the cell membrane structure, hence signaling, and epigenetics. Similar to FASN, other enzymes, such as the acetyl-CoA carboxylase ACC1/ACC2 system, are at the crossroad between several metabolic and functional pathways in cancer cells. Precisely identifying these nodal points may be used for future co-targeting anticancer strategies.

## Metabolic Plasticity in Metastatic Cascade

Metastasis remains a factor of poor prognosis for cancer patients and the main cause of death by cancer for which there is currently no specific treatment. In this session, speakers highlighted the key role of metabolic plasticity in controlling metastatic dissemination from the primary tumor to colonization of distant organs. Pierre Sonveaux (UCLouvain, Belgium) focused on monocarboxylate transporter 1 (MCT1), a proton/lactate cotransporter, that could contribute to the metastatic process and, therefore, constitutes an interesting target for metastatic prevention. He reported that MCT1 activates transcription factor NF-κB, which promotes cancer cell migration independently of MCT1 activity as a transporter. Unlike MCT1 activity inhibition, the downregulation of MCT1 expression inhibited migration, invasion and spontaneous metastasis *in vivo*. Paolo Ceppi (Uniklinikum Erlangen, Germany) then explained how glucose metabolism controls the epithelial-to-mesenchymal transition (EMT), a de-differentiation process used by cancer cells to acquire invasive and resistance properties. He identified that enzymes belonging to the polyol pathway (PP), aldo-keto reductase family 1 member B1 (AKR1B1), and sorbitol dehydrogenase, promote EMT and cell growth. The expression of these enzymes was correlated with poor patient survival. Bárbara Sousa (University of Porto, Portugal) described the control of breast cancer metabolism by the cell-cell adhesion molecule P-cadherin. She demonstrated that P-cadherin promotes glycolysis and reduces oxidative stress, suggesting an important role in anoikis resistance and metastasis. Altogether, these insights illustrated the crucial role of metabolic plasticity in cancer metastasis providing new opportunities of targeting.

## Tumor Heterogeneity and Metabolic Crosstalks

Tumors are known to be highly glycolytic, with lactate concentrations up to 40 mM in the tumor microenvironment. Hence, tumor metabolism is often considered as a homogeneous entity, with tumor relying only on aerobic glycolysis to survive and grow. This view is outdated as we are progressively acknowledging the impact of tumor heterogeneity on shaping metabolic fluxes. In particular, we are beginning to elucidate the network of “social” interaction between tumor cell populations, ranging from metabolic symbiosis to metabolic competition, based on the metabolic requirements of the ongoing biological activities and nutrient availability. The session dedicated to this topic provided several interesting points of discussion concerning tumor heterogeneity and metabolic crosstalks. For instance, Maria Yuneva (Francis Crick Institute, UK) discussed her work on metabolic reprogramming controlled by the MYC oncogene, highlighting the metabolic flexibility of tumors and defining potential vulnerabilities. Then, Maria Shoshan (Karolinska Institute, Sweden) presented her data investigating the impact of metabolic flexibility on cisplatin resistance. On a similar topic, the work of Giuseppe Taurino (University of Parma, Italy) concerned the importance of amino acid transport and stromal cooperation in counteracting nutritional stress in asparaginase-treated acute lymphoblastic anemia, thus evidencing the importance of such metabolic crosstalk in supporting cancer fitness. Tumor metabolic heterogeneity is not only shaped by cellular and biological heterogeneity, but it is also promoted by nutrient availability due to inefficient perfusion. To model this, Angela Otto (Technical University of Munich, Germany) presented her work concerning metabolic reprogramming under nutrient (glucose and glutamine) limitation *in vitro*. A major issue in assessing tumor metabolic heterogeneity is the limiting availability of tools to image metabolism *in vivo*. The talk of Valery Khramtsov (West Virginia University, USA) focused on the use of low-field electron paramagnetic resonance (EPR)-based techniques, in combination with specially designed functional paramagnetic probes, to measure various parameters such as oxygenation, pH, and inorganic phosphate availability, providing interesting observations on the correlation of specific nutrient availability with tumor aggressiveness.

## Tumor Acidosis and Metabolic Adaptations

This session was focused on intratumoral acidosis and its effect on cancer biology. Extracellular acidosis is mainly due to an enhancement of tumor-associated glycolytic activity also in conditions of normal oxygen supply (Warburg effect), and it is now considered to be a hallmark of cancer. As described by Dario Longo (University of Turin, Italy), it is currently possible to directly measure *in vivo* the intratumoral pH by using MRI-based approaches. This innovative technology will be crucial to measure intratumoral pH in real-time, both for diagnostic and prognostic purpose, as well as for the monitoring of treatment response, like for the evaluation of the cytotoxic effect of anticancer drugs that target proton pumps/transporters. Among these drugs, one example has been proposed by Ivana Novak (University of Copenhagen, Denmark) in pancreatic cancer that expresses both gastric and non-gastric H^+^/K^+^- ATPases. In this tumor type, H^+^/K^+^- ATPases contribute to secretin-stimulated secretion and participate in intracellular pH regulation. Their inhibition by using either proton pump inhibitors (PPI) or potassium-competitive blockers reduced tumor growth and dissemination. Similarly, as discussed by Stine Falsig Pedersen (University of Copenhagen, Denmark) in luminal breast carcinoma cells, targeting the Na^+^/H^+^ exchanger NHE1 and the Na^+^/${\text{HCO}}_{3}^{-}$ cotransporter NBCn1 delayed cell cycle progression in different ways, including the expression and phosphorylation of cell cycle regulatory proteins, with NHE1 mainly involved in the S phase and NBC1 in the G2/M phase. Karin Bartel (Ludwing-Maximilians-University of Munich, Germany) showed that, in addition to altering pH homeostasis, targeting proton/ion pumps and transporters might have an indirect effect on cancer cell metabolism. Indeed, inhibition of V-ATPase, a crucial regulator of lysosome acidification, produced a change in triacylglycerol distribution and composition, a different morphology in lipid droplets, and impaired mitochondrial functions. Also, MCTs regulate tumor metabolic alterations. As reported by Fatima Baltazar (University of Braga, Portugal), glioblastoma cells are forced to adopt a glycolytic phenotype in hypoxic tumor regions, with the production of high levels of both protons and lactate. In these cells, MCT1 targeting decreased glycolytic metabolism, cell migration, and invasion and induced cell death. Furthermore, as mentioned by Cyril Corbet (UCLouvain, Belgium), when coupled with the inhibition of mitochondrial pyruvate carrier, MCT1 targeting is even more cytotoxic and can be suggested for radiosensitization.

## Cancer Metabolism and Immune Responses

Interactions between cancer cells and their microenvironment are now recognized as major players in tumorigenesis, tumor growth, and shaping the evolutionary path of many if not all cancers. A large fraction of tumors display infiltration by immune cells, and intense research has been initiated to deconvolute the role of the immune system in the process. These interactions are, however, not unique to tumors, and insights in cancer metabolism and immune responses can be achieved by studying other *in vivo* processes where immune cells determine the fate of growing or regenerating tissues. In the opening talk, Massimiliano Mazzone (KULeuven, Belgium) showed intriguing data on how macrophages and skeletal muscle satellite cells compete for glutamine in the tissue environment. He provided evidence for this competition by showing that genetic deletion and pharmacologic targeting of glutamate dehydrogenase 1 (GLUD1) in macrophages leads to the rerouting of glutamate to glutamine synthesis and secretion supported by glutaminase 1 (GLS1) upregulation, which in turn sustains satellite cell activation, proliferation and differentiation, altogether accelerating muscle regeneration and functional recovery. In the following talks, two tumor-specific examples of metabolic pathways participating in immune-cancer cell crosstalk were presented by Stefano Biffo (University of Milan, Italy) and Maša Ždralević (Université Côte d'Azur, France and Centre Scientifique de Monaco, Monaco). First, Stefano Biffo has shown that a unique translational control of lipid synthesis enzymes (including ACC1) via eIF6 and eIF4E, operates in both cancer cells and immune responses, providing a potential adaptive framework for tumor evolution. Maša Ždralević presented further details on the mechanism of lactate secretion by cancer cells using cellular models with LDH-A/B depletion. She showed that knockout of both enzymes leads to re-activation of OXPHOS, which is sufficient to maintain tumor growth and renders tumor cells uniquely sensitive to OXPHOS inhibition. This raises the possibility that the primary role of lactate production by cancer cells is to provide signals to the tumor environment, including immune cells, as previously suggested. The closing talk by Mikhail M. Dikov (Ohio State University Medical Center, USA) reported exciting technological advances to assess these signals by profiling the tumor and lymph node metabolic microenvironment using EPR spectroscopy with a newly generated set of paramagnetic probes.

## Metabolic Targets in Cancer Therapy

Acquired treatment resistance is a predominant cause of treatment failure and death of cancer patients. Recent investigations showed that acquired resistance often reflects metabolic pathway rewiring. This session focused on novel treatment schemes co-targeting metabolic pathways upregulated as a consequence of treatment or tumor microenvironment conditions. Miguel Quintela-Fandino (CNIO, Spain) presented results from transcriptomic, phosphoproteomic and metabolomics studies exploring the combination of anti-angiogenic treatments with inhibitors of mitochondrial respiration in mouse models of lung and breast cancers. The group showed that small molecule anti-angiogenics led to vascular normalization associated with a metabolic shift toward OXPHOS, and that combination with e.g., metformin could induce metabolic synthetic lethality. On the other hand, monoclonal antibody anti-angiogenics exacerbated blood-vessel abnormality and hypoxia. This was associated with upregulation of a tumor-immune-suppressive phenotype, rendering such treatment synergistic with e.g., anti-PD-L1 treatment. Steven Hurstings (University of North Carolina, USA) combined a calorie restriction-mimetic diet (CR) with inhibition of autophagy, which is upregulated as a consequence of CR. Cell culture, as well as *in vivo* studies of pancreatic ductal adenocarcinoma (PDAC), revealed that growth-suppressive effects of CR in the form of rapamycin or IGF-1R/insulin receptor inhibition were strongly potentiated by co-treatment with chloroquine-related compounds to inhibit autophagy. There is, however, a serious lack of specific anti-autophagy treatments suitable for clinical use. Following up on this theme, Angelo de Milito (Sprint Bioscience, Sweden) presented data on the development and efficacy of inhibitors of class III phosphatidyl-3-kinase Vps34. Vps34 inhibition would be particularly powerful in combination with treatment schemes that elicit upregulation of autophagy. The team found that cytotoxicity of receptor tyrosine kinase inhibitors Sunitinib and Erlotinib in breast cancer cell lines was potentiated by Vps34, consistent with upregulation of autophagy by these treatments. Finally, Margherita Cortini (Rizzoli Orthopedic Institute, Italy) enlightened the crucial role of lipid metabolism in determining the aggressiveness of osteosarcoma in response to extracellular acidosis, with particular regard to the sphingolipid pathway.

## Conclusion

The somatic evolution of cancers leading to their invasive behaviors includes metabolic rewiring that endows cancer cells with the ability to survive and proliferate in a highly selective microenvironment of hypoxia, acidosis, and severe nutrient restriction. Over many months or years in this environment, cells emerge with more adaptable, aggressive and de-differentiated phenotypes. An important component of such phenotypes is aerobic glycolysis, known as Warburg effect. In the closing lecture, Robert J. Gillies (Moffitt Cancer Center, Tampa, USA) discussed the nature of the microenvironmental conditions and mechanisms that select for the glycolytic phenotype, highlighting work using an approach based on exposure of non-metastatic/non-glycolytic cancer cells to microenvironmental selection including acidity, hypoxia and low glucose followed by regrowth in normal conditions. Resulting clones exhibited increased rates of aerobic glycolysis and upregulation of a number of cancer-related genes. This approach explains why and how cells acquire a glycolytic phenotype in early cancers, which is a hallmark of aggressiveness and poor outcome.

ISCaM aims to promote interactions between basic and clinical researchers and between young and senior scientists, which was facilitated by the international composition of the meeting (80 attendees representing 14 countries), vivid discussions in the audience and beyond, and social activities. Oral and poster communications were demonstrated in sessions focusing on emerging aspects of cancer metabolism that were co-chaired by a principal and by a young investigator. Three young investigators (Jessica Whitburn, Preeta Anathanarayanan, and Sepideh Aminzadeh-Gohari) received travel grants from the Local Organizers. Prizes were awarded to young investigators who delivered best scientific presentations: the first ISCaM award for best oral presentation went to Elisabeth Wyart (her topic is highlighted above) and the second ISCaM award for the best poster presentation went to Joao Santiago de Jesus (UCLouvain, Belgium) for his study of acidosis-induced lipid metabolism in cancer invasiveness. In line with ISCaM's objective to improve the outcome of cancer patients through the discovery and development of new anticancer drugs, several potential anticancer strategies related to tumor metabolism were presented.

Conclusively, ISCaM2018 provided the audience with most recent discoveries on metabolic adaptations and targets in cancer. The follow up discussions will be held at the 6th annual ISCaM2019 meeting in Braga, Portugal, 19–21 October 2019, entitled “Cancer Metabolic Rewiring: Mapping the Road to Clinical Translations” (https://www.iscams.org/meeting/iscam2019-6th-annual-meeting-braga).

## Author Contributions

Authors were members of the Board of the International Society of Cancer Metabolism (ISCaM).

### Conflict of Interest

The authors declare that the research was conducted in the absence of any commercial or financial relationships that could be construed as a potential conflict of interest.
